# Hydrodynamics-based transfection of rat interleukin-10 gene attenuates porcine serum-induced liver fibrosis in rats by inhibiting the activation of hepatic stellate cells

**DOI:** 10.3892/ijmm.2014.1831

**Published:** 2014-07-02

**Authors:** YUE-HONG HUANG, YUN-XIN CHEN, LI-JUAN ZHANG, ZHI-XIN CHEN, XIAO-ZHONG WANG

**Affiliations:** Department of Gastroenterology, Fujian Medical University Union Hospital, Fuzhou, Fujian 350001, P.R. China

**Keywords:** interleukin-10, gene therapy, liver fibrosis, hydrodynamics-based transfection, hepatic stellate cells, co-culture

## Abstract

Liver fibrosis is the common pathological outcome for the majority of chronic liver diseases. Interleukin-10 (IL-10) is a cytokine that downregulates proinflammatory responses and has a modulatory effect on liver fibrogenesis. However, little is known regarding the effect of rat interleukin-10 (*rIL-10*) gene by hydrodynamics-based transfection (HBT) on liver fibrosis in rats. The aim of this study was to investigate the effect of the *rIL*-*10* gene by HBT on the progression of liver fibrosis induced by porcine serum (PS) in rats and explore its possible mechanism. Plasmid-expressing rIL-10 was transferred into rats by HBT and immunohistochemistry and RT-PCR were used to detect the major organ expressing rIL-10. Liver fibrosis was induced in rats by intraperitoneal administration of PS for 8 weeks. Plasmid pcDNA3-rIL-10 solution was administered weekly by HBT starting at the 5th week. Liver function and hepatic histology were examined. The possible molecular mechanisms of *rIL*-*10* gene therapy were assessed in liver tissue and hepatic stellate cells (HSCs) co-cultured with BRL cells (a hepatocyte line) *in vitro*. The results showed rIL-10 expression occurred mainly in the liver following *rIL*-*10* gene transfer by HBT. Maintaining a stable expression of rIL-10 in serum was assessed by repeated administration. The *rIL*-*10* gene treatment attenuated liver inflammation and fibrosis in PS-induced fibrotic rats, reduced the deposition of collagen and the expression of α-smooth muscle actin (α-SMA) in fibrotic rats. The *in vitro* experiment showed that the expression of a-SMA and procollagen type I in HSCs co-cultured with the BRL-transfected *rIL*-*10* gene were significantly decreased. These findings indicate that *rIL*-*10* gene therapy by HBT attenuates PS-induced liver fibrosis in rats and that its mechanism is associated with rIL-10 inhibiting the activation of HSCs and promoting the degeneration of collagen.

## Introduction

Liver fibrosis is the response of the liver to diverse chronic insults such as chronic viral infection (mostly hepatitis B and C virus), parasitic disease and toxic damage (1). Liver fibrosis is characterized by the activation of hepatic stellate cells (HSCs), which are then involved in the synthesis of matrix proteins and the regulation of matrix degradation (2). The advanced stage of fibrosis is cirrhosis. Liver cirrhosis, the irreversible terminal stage of chronic liver disease, is a major cause of morbidity and mortality worldwide, with no effective therapy (3). Many factors are involved in the development of fibrosis, including profibrotic cytokines, chemokines, eicosanoids, fibrinolytic/fibrinogenic factors, matrix metalloproteinases and their inhibitors, and oxidative stress (4).

Interleukin-10 (IL-10) is a cytokine that downregulates the proinflammatory response and has a modulatory effect on hepatic fibrogenesis (5–7). IL-10 has a direct effect on the production of collagen and collagenases and modulates remodeling of the extracellular matrix (ECM) (8). Results of previous studies have shown that IL-10 may be important in antifibrogenesis during CCl4-induced hepatic fibrogenesis (9–11). However, the half-life of IL-10 is extremely short, at approximately 2–3 h. Maintaining a stable expression in the serum is difficult. Gene therapy is expected to resolve this problem and may be a useful method for the early stage of liver cirrhosis. Gene therapy may prove successful following the development of effective gene delivery systems. An ideal vector would be able to deliver genetic material efficiently, and would result in a high-level, properly-regulated and prolonged expression. Additionally, this vector would be non-toxic, non-immunogenic, and have a broad host range (12). It has been reported that high levels of foreign gene expression in murine hepatocytes can be achieved by the rapid injection of a large volume of a naked DNA solution into the tail vein. This process is known as ‘hydrodynamics-based transfection (HBT)’ (13,14). The aim of the present study was to evaluate the anti-fibrotic effects of the rat interleukin-10 (*rIL-10*) gene by HBT gene delivery system on experimental liver fibrosis in rats and its possible mechanism.

## Materials and methods

### Rats

Clean male Sprague-Dawley rats weighing 100–120 g were provided by the Shanghai Experimental Animal Center (Shanghai, China). The rats were bred at a room temperature of 22±2°C, humidity of 55±5%, with a 12-h alternating light/dark cycle and access to water and food *ad libitum*. The feed was provided by the BK Company (Shanghai, China). Animal procedures were performed under the control of the animal care committee of Fujian Medical University in accordance with the Guidelines on Animal Experiments in Fujian Medical University.

### Plasmid DNA preparation

Large-scale plasmid DNA preparation was produced using the alkaline lysis method (Qiagen, Beijing, China; no. 12362, USA). The plasmid preparation for *in vivo* injections was suspended in sterile deionized water.

### Intravenous injection of plasmid DNA

Injection of plasmid DNA was performed as described by Liu *et al* and Zhang *et al* (13,14). Briefly, plasmid DNA (1.4 μg/g) in lactated Ringer’s solution (0.1 ml/g body weight) was injected into the tail vein. The DNA injection was completed in 10–15 sec. With this delivery system, the plasmid was trapped in the liver where it produced cytokine which was then transported into the bloodstream and perfused the organs (15,16).

### Animal model and experimental protocols

Twenty-seven Sprague-Dawley rats with a body weight of 100–120 g were used, and experiments were performed in accordance with the institutional ethics guidelines of the Fujian Medical University Union Hospital. Hepatic fibrosis was induced by intraperitoneal injections of 0.5 ml porcine serum (PS) (PAA Laboratories, Linz, Austria) twice a week for 8 weeks. Control rats (CTRL) were injected 0.5 ml physiological saline twice a week by intraperitoneal injection. From the 5th week, fibrotic rats were randomly divided into 3 groups: fibrotic rats injected weekly with Ringer’s solution through the caudal vein (PS), or rIL-10 recombinant plasmid DNA (PS-pcDNA3-rIL-10), or PcDNA3 empty vector (PS-pcDNA3). At the end of the 8th week, the experimental rats were sacrificed under anesthesia of 10% chloral hydrate.

### Histopathological examination

Liver of rats receiving a rIL-10 plasmid DNA (pcDNA3-rIL-10) injection on days 1, 7 and 14 following the gene transfer and liver of fibrotic rats at the end of the 8th week were harvested. The samples were fixed in 10% formalin and embedded with paraffin. Sections were stained with hematoxylin and eosin (H&E) and evaluated by two pathologists.

### Sirius red staining and collagen measurement

The sections were deparaffinized with xylene and rehydrated with graded ethanol. After rinsing the sections with distilled water 3 times, the sections were stained in 0.1% Sirius red in saturated picric acid solution for 30 min, and placed in ethanol for differentiation for 2 min. The sections were then rinsed in phosphate-buffered saline once and water twice for 30 sec each to remove any unbound dye. After drying for 2 h, the slides were mounted. The quantitative analysis of collagen type I and III was carried out using the Olympus-BX41 image analyzing system in five microscopic fields (magnification, x40) per section. The average of the five fields was calculated for assessment of the degree of fibrosis in each case. All the sections were examined by the same pathologist who was blind to the experimental design. The liver tissue was distinguished from the background according to a difference in light density. This allowed the measurement of the total liver tissue area. The amount of connective tissue, stained red, was then measured. Subsequently, the percentage of collagen on the section was measured.

### Liver function assays

Serum levels of aspartate aminotransferase (AST) and alanine aminotransferase (ALT) were measured by routine methods in the clinical laboratory of our institution.

### ELISA assay

Serum samples and culture supernatant were assayed for rIL-10 using an ELISA kit according to the manufacturer’s instructions (Biosource International, Inc., Camarillo, CA, USA).

### RT-PCR assay

Total RNA was extracted using TRIzol reagent (Gentra Systems, Inc., Minneapolis, MN, USA), and then reverse transcribed to cDNA according to the instructions of the MMLV reverse transcription kit (Promega, Madison, WI, USA). Using 2 μl RT products as the template, the PCR reaction contained 10 pmol for each primer (rIL-10, sense: 3′-cgaagcttgccaccatgcttggctcagcac-5′ and antisense: 3′-cgtcta gatcaatttttcattttgagtg-5′, product, 559 bp; β-actin, sense: 3′-ccaaccgtgaaaagatgacc-5′ and antisense 3′-caggaggagcaa tgatcttg-5′, product, 660 bp to detect the rIL-10 mRNA expression in rat liver, lung, heart, kidney and spleen tissue. The samples were placed in a thermocycler with the incubation program at 95°C for 5 min, then 30 cycles at 94°C for 45 sec, at 61°C for 30 sec, and an extension at 72°C for 1 min. Products of RT-PCR were electrophoresed on a 16 g/l agarase gel to reveal the amplified bands.

### Immunohistochemistry

Rat liver tissues were sectioned at a thickness of 4 μm. Following deparaffinization with xylene and dehydration with graded ethanol, the sections were incubated in PBS containing 30 ml/l H_2_O_2_ to remove endogenous peroxidase and in PBS containing 0.1 mol/l citrate to retrieve microwave antigens and then incubated with normal goat serums to block the non-specific binding sites. Following incubation with primary antibodies against rIL-10 (Biosource International, Inc.), α-SMA (Abcam, Hong Kong, China), the sections were treated with instant S-P immunohistochemical reagents (Zhongshan Golden Bridge Biotechnology Co., Ltd., Beijing, China) and incubated in a buffer solution containing 3,3′-diaminobenzidine tetrahydrochloride (DAB) and H_2_O_2_ to produce a brown reaction product. The sections were then dehydrated and coverslipped. Microscopic examination of the sections was performed and the results were assessed as previously described (9).

### BRL cell culture and transfection

BRL cells, an immortalized normal rat hepatocyte line obtained from the cell bank of Academia Sinica, Shanghai, China, were seeded in 30-ml plastic flasks at 1x10^6^ cells/well and cultivated in DMEM supplemented with 10% fetal bovine serum (FBS) in a humidified incubator containing 5% CO_2_ at 37°C. When cell density reached 60–70% confluence, transfection was performed according to the manufacturer’s instructions (Polyplus Transfection, New York, NY, USA). BRL cells were separately transfected with plasmid pcDNA3-rIL-10, pcDNA3 and normal saline for 24 h using JetPEI™-Hepatocyte DNA transfection reagent (Polyplus Transfection, New York, NY, USA). The transfection system contained 5 μg DNA and 16 μl JetPEI-Hepatocyte per flask.

### HSC culture

HSCs, an immortalized normal rat HSCs line obtained from the cell bank of Academia Sinica, were seeded in 6-wells at 3x10^5^ cells/well in 2 ml DMEM containing 10% FBS the day prior to BRL cell transfection.

### Co-culture of HSC and BRL cells

Following transfection for 24 h, BRL cells were digested and seeded in 30-mm filter inserts (Millipore, Billerica, MA, USA) and placed in 6-well culture plates at 2x10^5^ cells/insert in 3 ml DMEM containing 10% FBS for 3 h. Inserts planted with BRL were placed in the 6-well plastic tissue culture plates which had been planted with HSCs and the culture medium was then refreshed. The co-culture cells were divided into HSCs co-cultured with BRL transfected with i) normal saline (HSCs/BRL); ii) pcDNA3.0 (HSCs/BRL-rIL-10^−^); and iii) pcDNA3.0-IL-10 (HSCs/BRL-rIL-10^+^). Co-culture was terminated after 48 h.

### Western blotting

After 48-h co-culture, HSCs were washed with PBS twice and lysed with cell lytic buffer containing 50 mM Tris pH 8.0, 150 mM NaCl, 0.2 mg/ml NaN3, 1 mg/ml SDS, 0.1 mg/ml aprotinin, 10 mg/ml NP-40, and 5 mg/ml sodium deoxycholate and 0.1 mg/ml phenyl-methylsulfonyl fluoride. The supernatants were obtained following centrifugation at 1,500 x g for 10 min. The protein concentrations of the cytosolic extracts were determined by the Bradford protein assay. Equal amounts of protein were separated on a 12% SDS-polyacrylamide gel and transferred onto nitrocellulose membranes. Monoclonal antibodies of procollagen type I, α-SMA (Abcam) and β-tubulin (Santa Cruz Biotechnology, Inc., Santa Cruz, CA, USA), respectively, were used at a dilution of 1:50, 1:400 and 1:150, respectively, followed by incubation with the homologous secondary antibody labeled with HRP (Santa Cruz Biotechnology, Inc.). The signals were visualized using an ECL kit.

### Statistical analysis

Data are expressed as the means ± SD. The significance for the difference between the groups was studied using one-way ANOVA with SPSS 13.0 software. P<0.05 was considered to indicate statistical significance.

## Results

### Distribution of rIL-10 mRNA following rIL-10 gene by HBT

**Figure 1:**
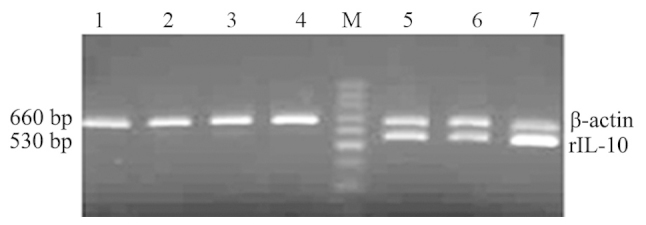
Distribution of rat interleukin-10 (rIL-10) mRNA after *rIL*-*10* gene by hydrodynamics-based transfection (HBT). Lanes 1) heart; 2) lung; 3) kidney; 4) spleen; 5) day 14 liver; 6) day 7 liver; 7) day 1 liver; M) marker.

An effective liver-targeted gene delivery system is crucial for a successful gene therapy in liver disease. Therefore, in this study, the *rIL*-*10* gene was used as a reporter gene. RT-PCR was used to detect the expression of rIL-10 and control β-actin mRNA in liver, spleen, kidney, heart and lung after PcDNA3-rIL-10 plasmid DNA was transferred by HBT. The results showed a positive expression of rIL-10 mRNA was observed mainly in the liver on day 1 after transfection and the expression of rIL-10 mRNA in the liver was sustained for two weeks after single hydrodynamic injection (Fig. 1).

### Protein expression of rIL-10 in the liver after rIL-10 gene by HBT

**Figure 2:**
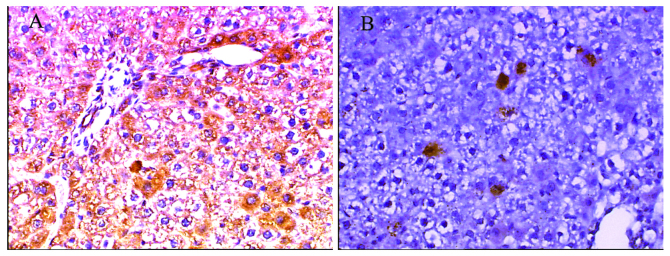
Protein expression of rat interleukin-10 (rIL-10) in the liver after *rIL-10* gene by hydrodynamics-based transfection (HBT). Expression on (A) day 1 and (B) day 7.

To confirm rIL-10 expression mainly in the liver after gene transfer, we detected the protein expression of rIL-10 in the liver, spleen, kidney, heart and lung after *rIL*-*10* gene via HBT on days 1 and 7 by immunohistochemical analysis. The results showed that a positive expression of rIL-10 was detected mainly in hepatocytes, with ~70% hepatocytes expressing rIL-10 protein on day 1 (Fig. 2A), while only some positive expression was detected in kidney. However, no positive expression after *rIL*-*10* gene was transferred for 24 h (data not shown) was detected in the spleen, heart and lung tissue. The number of positively stained cells was decreased on day 7 after *rIL*-*10* gene transfer (Fig. 2B). Results of the semi-quantitative analysis revealed that the expression of rIL-10 on day 1 was 9-fold higher (9165.98±2031.71) than that on day 7 (1047.94±69.00).

### Levels of rIL-10 in the serum after rIL-10 gene by HBT

**Figure 3:**
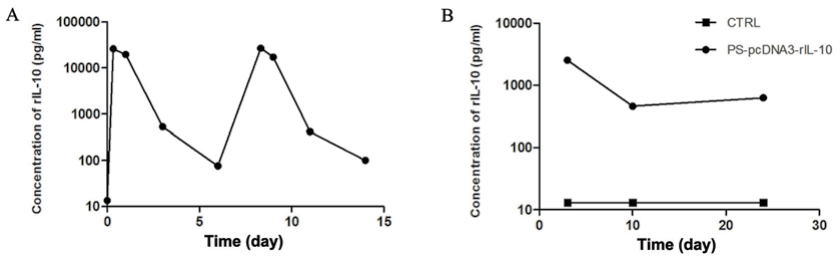
Levels of rat interleukin-10 (rIL-10) in the serum. (A) Levels of rIL-10 after rIL-10 gene repeating injection. (B) Levels of rIL-10 in fibrotic rats treated with rIL-10 gene.

Maintaining a stable gene expression *in vivo* for the gene therapy of liver fibrosis was crucial. Therefore, ELISA was assayed to detect the levels of rIL-10 in serum. As shown in Fig. 3A, the time-response curve showed levels of rIL-10 in the serum reached a peak level ~8 h after *rIL*-*10* gene transfer and subsequently decreased. However, the peak level of rIL-10 expression was regained by repeated injection of rIL-10 plasmid DNA.

To examine the effect of *rIL*-*10* gene treatment on liver fibrosis induced by PS in rats, we detected the rIL-10 expression in serum of PS-induced fibrotic rats on day 3 after *rIL*-*10* gene treatment. The results showed that levels of rIL-10 maintained high levels in the *rIL*-*10* gene-treated rats (Fig. 3B).

### Effect of plasmid transfer by HBT on hepatic function

To determine whether the gene transfer procedure caused any adverse effects in rats, we detected the serum concentration of AST and ALT. Rats were assigned to two groups: one group of rats received an injection of Ringer’s solution containing pcDNA3 null plasmid, while the other group of rats only received an injection of Ringer’s solution. The ALT serum of pcDNA3 and Ringer’s solution rats was significantly increased on day 1 after the injection, and subsequently returned to normal levels by day 3, and maintained to day 7. Similar results regarding AST serum levels were observed in the two groups (Table I).

### The therapeutic effects of rIL-10 gene treatment by HBT on liver fibrosis in rats

**Figure 4:**
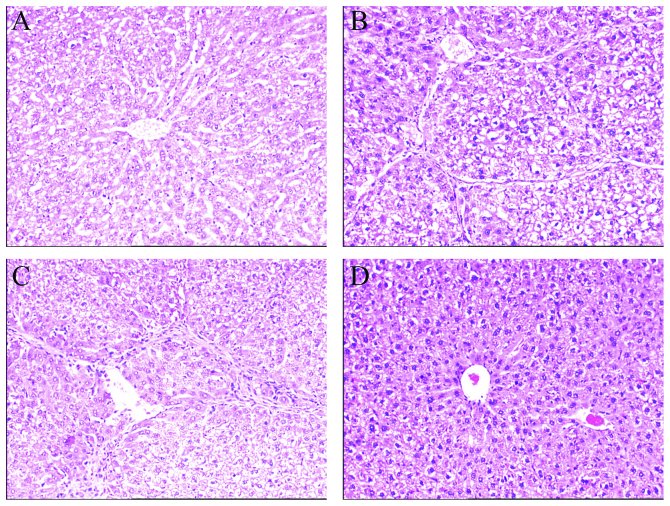
The therapeutic effects of rat interleukin-10 (*rIL-10*) gene treatment on liver fibrosis in rats. (A–D) Photomicrographs of liver tissue by hematoxylin and eosin (H&E) (magnification, x200) to show hepatic fibrosis in (A) normal control rats (CTRL) or (B) porcine serum (PS)-induced fibrotic rats treated with saline or (C) with pcDNA3 or (D) with pcDNA3-rIL-10.

The results of our experiment showed that high levels of *rIL*-*10* gene expression in rat hepatocytes can be achieved by HBT. To confirm the therapeutic effect of liver targeting rIL-10 expression on liver fibrosis, we performed a second experiment. The liver fibrosis model was induced by PS, with rats being treated with *rIL-10* gene by HBT from the 4th week. Liver biopsy is the gold-standard method for detecting changes in liver fibrosis. Fig. 4 shows representative histological changes in the liver of different groups. The H&E staining showed that advanced fibrosis was established after 8-week administration of PS. Rats in group CTRL (Fig. 4A) showed normal lobular architecture with central veins and radiating hepatic cords. Rats in group PS (Fig. 4B) and group PS-pcDNA3 (Fig. 4C) showed a slight chronic inflammatory infiltrate in the portal area, scattered necrotic and regenerative hepatocytes, and marked increase in ECM content, which resulted in large fibrous septa and distorted tissue architecture, and formed abnormal hepatic lobules. Rats in group PS-pcDNA3-rIL-10 (Fig. 4D) showed a marked reduction of inflammation, hepatocyte damage and deposition of collagen fibers.

To determine the degree of necro-inflammatory liver injury and fibrosis, histological grading and quantification were blindly performed by the pathologist. The grade of inflammation and stage of fibrosis in liver were markedly decreased in group PS-pcDNA3-rIL-10 compared to groups PS and PS-pcDNA3 (p<0.01). No significant differences were observed in the grade of inflammation and stage of fibrosis between groups PS and PS-pcDNA3 (p>0.05; Table II).

### Effects of rIL-10 gene treatment on the hepatic function in fibrotic rats

**Figure 5:**
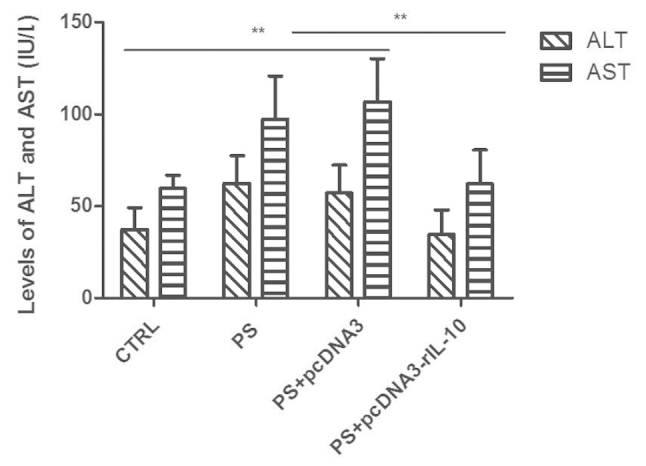
Hepatic function in different groups in fibrotic rats after rat interleukin-10 (*rIL*-*10*) gene treatment. Data shown are expressed as the means ± SD from 6–7 rats per group. ^**^P<0.01.

At the end of the experiment, an analysis of ALT and AST serum was carried out to evaluate the amount of liver injury in fibrotic rats. The results showed that levels of ALT and AST in group PS and PS-pcDNA3 were significantly higher than those in group CTRL (p<0.01). Compared to group PS and PS-pcDNA3, levels of ALT and AST evaluated in group PS-pcDNA3-rIL-10 were significantly decreased (p<0.01). No significant differences were observed in ALT and AST between group CTRL and group PS-pcDNA3-rIL-10 (p>0.05; Fig. 5).

### The effects of rIL-10 gene treatment on the deposition of collagen in fibrotic rats

**Figure 6:**
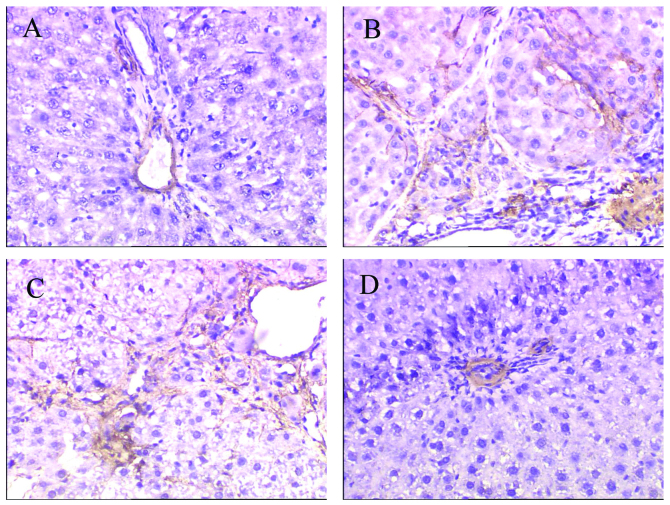
Effects of rat interleukin-10 (*rIL*-*10*) gene treatment on the deposition of collagen in fibrotic rats. Photomicrographs of liver tissue by Sirius red staining (magnification, x200) to show collagen deposition in (A) normal control rats (CTRL) and (B) in porcine serum (PS)-induced fibrotic rats treated with saline or (C) with pcDNA3 or with (D) pcDNA3-rIL-10.

**Figure 7:**
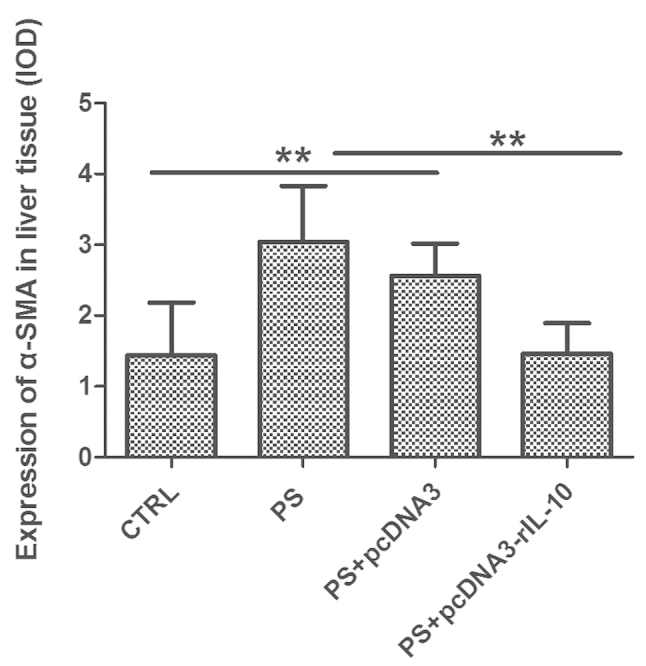
Relative expression of collagen types I and III in liver tissue. Data shown are expressed as the means ± SD from 6–7 rats per group. ^**^P<0.01.

One of the key characteristics of liver fibrosis is the excessive deposition of collagen in the liver, especially collagen types I and III. Sirius red staining was used to detect the deposition of collagen. Collagen types I and III were stained intensely red with the Sirius red staining, while the non-collagen tissue was stained yellow. PS treatment for 8 weeks induced a significant deposition of collagen types I and III (Fig. 6B), which developed a severe fibrosis, compared with the group CTRL (Fig. 6A). Similar results were observed in the group PS-pcDNA3 (Fig. 6C). Following *IL*-*10* gene treatment for 4 weeks, the deposition of collagen types I and III was markedly reduced (Fig. 6D). Results of the semi-quantification analysis of the area of collagen types I and III revealed that the collagen content in group PS increased 9-fold compared with group CTRL (p<0.01). No significant differences were detected in the collagen content between group PS and PS-pcDNA3 (p>0.05), while the areas of collagen were markedly decreased in the group PS-pcDNA3-rIL-10 (p<0.01; Fig. 7).

### Effects of rIL-10 gene treatment on the HSC activation in fibrotic rats

**Figure 8:**
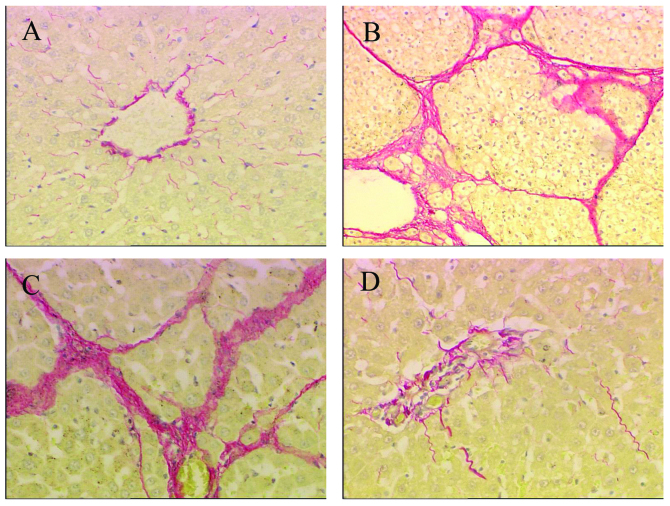
Effects of rat interleukin-10 (*rIL*-*10*) gene treatment on HSC activation. α-SMA-positive expression in liver by immunohistochemistry (magnification, x200) in (A) normal control rats (CTRL) and in (B) porcine serum (PS)-induced fibrotic rats treated with saline or (C) with pcDNA3 or (D) with pcDNA3-rIL-10.

**Figure 9:**
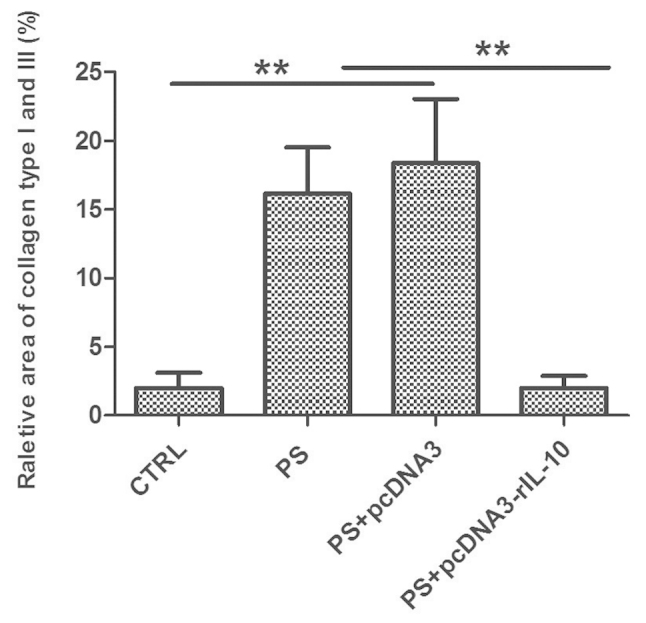
Relative expression of α-SMA in liver tissue. Data shown are expressed as the means ± SD from 6–7 rats per group. ^**^P<0.01.

The activated HSCs are a rich source of ECM proteins such as collagen type I and fibronectin (17). To determine the effect of *rIL*-*10* gene treatment on liver fibrosis in rats by inhibiting the activation of HSC, we measured the expression of the HSC activation marker α-SMA by immunohistochemical analysis. The results revealed that a positive expression of α-SMA was localized only in vascular smooth muscle cells in CTRL rats (Fig. 8A), while the distribution of α-SMA-positive cells in group PS was similar to that of deposition of collagen. α-SMA-positive cells were significantly increased in the liver of PS and PS-pcDNA3 rats compared with CTRL rats (Fig. 8B–C), while the *rIL*-*10* gene treatment reduced the number of α-SMA-positive cells in PS-induced rats (Fig. 8D). Semi-quantitative analysis revealed that the expression of α-SMA was markedly higher in groups PS and PS-pcDNA3 as compared to group CTRL (p<0.01), while the expression of α-SMA in group PS-pcDNA3-rIL-10 was significantly reduced compared to group PS (p<0.01; Fig. 9).

### The expression of procollagen type I and α-SMA in HSCs co-cultured with BRL cells transfected with rIL-10 gene

**Figure 10:**
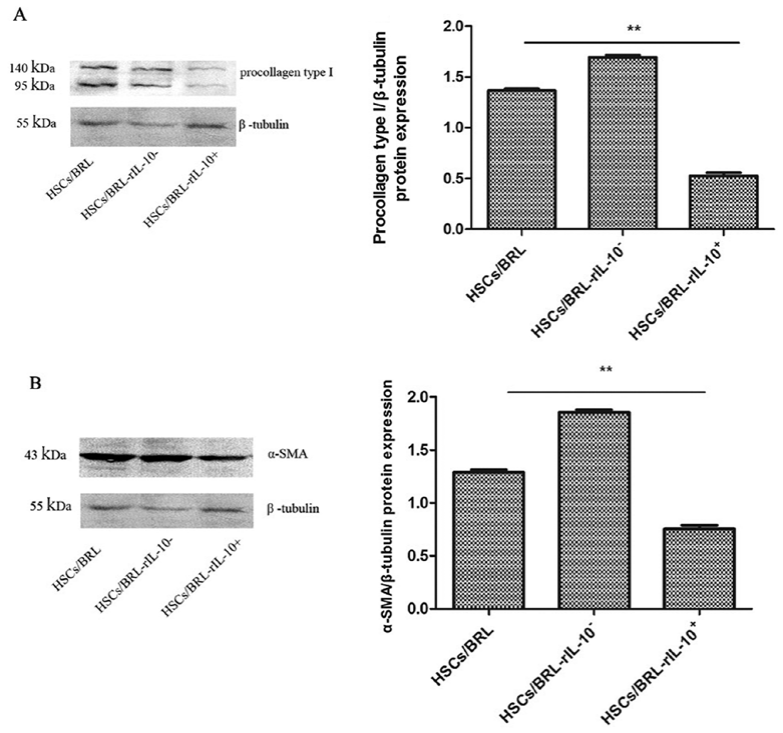
Expression of procollagen type I and α-SMA in co-culture hepatic stellate cells (HSCs). (A) Expression of procollagen type I; (B) expression of α-SMA. HSCs/BRL: HSCs co-cultured with BRL-transfected saline; HSCs/BRL-rat interleukin-10^−^ (rIL-10^−^): HSCs co-cultured with BRL-transfected pcDNA3; HSCs/BRL-rIL-10^+^: HSCs co-cultured with BRL-transfected pcDNA3-rIL-10. Data are expressed as the means ± SD. ^**^P<0.01.

To confirm *in vivo* the therapeutic effects of hepatocyte targeting expression rIL-10 on liver fibrosis, *in vitro* we examined the expression of procollagen type I and α-SMA in HSCs co-cultured with BRL cells transfected with rIL-10 expression plasmid pcDNA3-rIL-10, null plasmid pcDNA3 or saline. Results of the western blot analysis revealed that the expression of procollagen type I was significantly decreased in group HSCs/BRL-rIL-10^+^ compared with the control group HSCs/BRL-rIL-10^−^ and HSCs/BRL (p<0.01; Fig. 10A). No significant differences were detected in the levels of procollagen type I between groups HSCs/BRL-rIL-10^−^ and HSCs/BRL (p>0.05). The expression of α-SMA in HSCs was detected in the three groups by western blotting, while the expression of α-SMA in group HSCs/BRL-rIL-10^+^ was significantly decreased compared to the control group HSCs/BRL-rIL-10^−^ and HSCs/BRL (p<0.01; Fig. 10B).

## Discussion

The present study has demonstrated that *rIL*-*10* gene transfer by HBT attenuates PS-induced liver fibrosis in rats, while the mechanism was associated with the hepatocytes targeting the expression of cytokine rIL-10 via paracrine action, thereby inhibiting the activation of HSCs and promoting degeneration of collagen type I .

IL-10 was initially discovered in 1989, as a cytokine synthesis inhibitory factor for T lymphocytes (18). IL-10 is a pleiotropic cytokine, and one of the most important properties of IL-10 is its anti-inflammatory inhibitory action which restrains the immune response under various stimuli (8). Results of previous studies have shown that IL-10 exerts antifibrotic effects on fibrosis in rats (19,20). The study by Wang *et al* has shown that IL-10 mRNA and protein levels were increased in early liver fibrosis and disappeared in advanced liver fibrosis (6). Previously, we showed that exogenous cytokine IL-10 suppresses liver fibrosis progression induced by CCL4 in rats (9). However, recombinant cytokine IL-10 has short half-lives, necessitating frequent administration (21). To overcome this problem, a gene therapy system providing a continuous delivery may be more effective than an intermittent one. The most important factor for successful gene therapy of liver disease is to express the relevant gene effectively in the living organisms. Among the various gene delivery systems, the virus vector is laborious and expensive and may induce an immune response and lead to side effects that restrict repeated administration thereof (22). However, gene delivery based on plasmid DNA seems to be the simplest and safest strategy. It has been reported that high levels of foreign gene expression in murine hepatocytes can be achieved by the rapid injection of a large volume of naked DNA solution into the tail vein (22). This is known as ‘HBT’ (15), however, whether this gene delivery system is suitable for rats remains to be determined. Results of a previous study have confirmed that death of rats was markedly increased when the volume of Ringer’s solution reached 100 ml.kg^−1^ (data not shown). Thus, in this study, a volume of 80 ml.kg^−1^ of Ringer’s solution containing rIL-10 expression plasmid (pcDNA3-rIL-10) was rapidly injected into rat by HBT. The results showed that the liver is the major organ and hepatocytes are the major cells involved in *rIL*-*10* gene expression, while rIL-10 serum levels markedly increased following the transfer for 8 h. High levels of rIL-10 were sustained for at least 1 week in the serum and hepatocyte, while the rIL-10 mRNA expression in liver was sustained for 2 weeks following a single injection, which is in agreement with a previous study (23). AST and ALT serum is excreted from liver tissue into the circulation in proportion to the degree of hepatocyte damage, and the levels are thought to be one of the most sensitive markers of liver injury and disease progression (24). Therefore, we detected the serum concentration of AST and ALT to assess the security of gene transfer by HBT. The data showed that AST and ALT levels retained normal level after injection for 3 days. These results demonstrate that rIL-10 transfer into the liver using the HBT system maintained a sustained high-level expression of rIL-10 in rats. Thus this technique has the potential to be applicable to the treatment of liver disease, especially liver fibrosis.

Most hepatic fibrosis models are established by inducing so-called post-necrotic hepatic fibrosis. However, the PS-induced hepatic fibrosis model is characterized by minor hepatocyte damage but intense immune response. Given the chronic administration of the heterogeneous serum (25), the mechanisms of fibrogenesis are similar to those of hepatic disease in human (26), especially viral hepatitis. Therefore we used PS-induced liver fibrosis to mimic immune pathogenesis in human. The fibrotic rats received the *rIL-10* gene treatment by HBT starting from the 4th week. Liver biopsy is the gold-standard method for detecting changes in liver fibrosis (27). H&E staining results showed that PS-induced fibrotic rats exhibited a marked increase in ECM content, which resulted in large fibrous septa and distorted tissue architecture, and formed abnormal hepatic lobules, although hepatocyte damage was slight, as observed in findings of a recent study (28). Hepatocyte necrosis, deposition of collagen, the score of fibrosis and AST and ALT serum levels were markedly reduced in the *rIL*-*10* gene treatment for 4 weeks. The results suggest that liver targeting expression of *rIL-10* gene showed significant therapeutic effects for PS-induced liver fibrosis in rats.

During fibrotic progression, inflammation and liver injury trigger complex cell events that result in collagen deposition and the disruption of normal liver architecture. Activated HSCs are considered the most important cell type for the production of collagens (29). The PS-induced rat liver fibrosis is also caused by activated HSCs (27). During activation, HSCs transit into myofibro-blast-like cells that express α-SMA and these activated HSCs excrete ECM proteins, especially collagen types I and III, in hepatic fibrosis (30). α-SMA and collagen type I are useful biomarkers for the assessment of the therapeutic efficacy of the *rIL*-*10* gene treatment. The present results show that *rIL*-*10* gene treatment by HBT potently inhibited the α-SMA expression and significantly decreased the deposition of collagen types I and III in fibrotic rats liver. The present study confirmed that hepatocytes are the main cell involved in rIL-10 expression after gene transfer by HBT. We suggest that the therapeutic effects of *rIL*-*10* gene treatment by HBT are possible through rIL-10 paracrine hepatocytes to affect the activation of HSCs and the deposition of ECM. To confirm that the *rIL*-*10* gene in hepatocyte targeting expression inhibits the activation of HSCs and reduces the deposition of ECM, *in vitro* HSCs co-cultured with BRL cells were used to transfect the *rIL*-*10* gene or control null plasmid or saline. The results showed that the expression of procollagen type I and α-SMA in HSCs co-cultured with BRL-transfected *rIL*-*10* gene was significantly lower than the expression of the saline and null plasmid control groups. These data *in vitro* suggest that *rIL*-*10* gene in hepatocyte targeting expression can inhibit the activation of HSCs and promote the degeneration of collagen and confirm that hepatocyte targeting gene delivery may be an ideal technique for the *IL*-*10* gene therapy of liver fibrosis.

In conclusion, the study *in vivo* and *in vitro* suggests that HBT of the *rIL*-*10* gene ameliorates the degree of PS-induced liver fibrosis in rats, while its mechanisms may involve inhibition of the activation of HSCs and promotion of the degeneration of collagen.

## Figures and Tables

**Table I:** Effect of plasmid transfer by HBT on hepatic function.

	ALT		AST	
		
Groups (day)	Ringer’s solution	Plasmid DNA solution	Ringer’s solution	Plasmid DNA solution
−1	44.5±8.83		68.33±11.55	
1	202.33±59.33^b^	228.5±30.34^b^	428.5±121.30^b^	451.33±118.19^b^
3	45.17±17.22	40.83±11.41	77.17±13.21	94.00±30.29^a^
7	34.83±11.03	38.50±13.52	65.00±15.05	72.50±21.22

HBT, hydrodynamics-based transfection; AST, aspartate aminotransferase; ALT, alanine aminotransferase. Data shown are expressed as the means ± SD from 5 rats per group.

b p<0.01: vs. day -1;

a p<0.05: vs. day -1.

**Table II:** The grading and staging of HAI.

		Grading of inflammation					Staging of fibrosis				
			
Groups	Nos.	0	1	2	3	4	0	1	2	3	4
CTRL	6	6	0	0	0	0	6	0	0	0	0
PS^a^,^c^	7	0	2	5	0	0	0	0	0	2	5
PS + pcDNA3^a^,^c^	6	0	1	5	0	0	0	0	0	2	4
PS + pcDNA3-rIL-10^b^,^d^	7	4	3	0	0	0	0	6	1	0	0

HAI, rat liver histopathology; CTRL, normal control rats; PS, porcine serum-induced fibrotic rats treated with saline; PS + pcDNA3, porcine serum-induced fibrotic rats treated with pcDNA3; PS + pcDNA3-rIL-10, porcine serum-induced fibrotic rats treated with pcDNA3-rIL-10. Grading of inflammation:

a P<0.01, compared to group CTRL;

b P<0.01, compared to groups PS and PS + pcDNA3. Staging of fibrosis:

c P<0.01, compared to group CTRL;

d P<0.01, compared to groups PS and PS + pcDNA3.
